# Abnormal Red Cell Structure and Function in Neuroacanthocytosis

**DOI:** 10.1371/journal.pone.0125580

**Published:** 2015-05-01

**Authors:** Judith C. A. Cluitmans, Carlo Tomelleri, Zuhal Yapici, Sip Dinkla, Petra Bovee-Geurts, Venkatachalam Chokkalingam, Lucia De Franceschi, Roland Brock, Giel J. G. C. M. Bosman

**Affiliations:** 1 Department of Biochemistry, Radboud Institute for Molecular Life Sciences, Radboud University Medical Center, Geert Grooteplein 28, 6525 GA, Nijmegen, The Netherlands; 2 Department of Medicine, University of Verona-AOUI-Verona, Policlinico GB Rossi, P.le L Scuro, 10, 37134 Verona, Italy; 3 Division of Child Neurology, Department of Neurology, Istanbul Faculty of Medicine, Istanbul University, Istanbul, Turkey; 4 Department of Physical Organic Chemistry, Radboud University Nijmegen, Institute for Molecules and Materials, Heyendaalseweg 135, 6525 AJ, Nijmegen, The Netherlands; Université Claude Bernard Lyon 1, FRANCE

## Abstract

**Background:**

Panthothenate kinase-associated neurodegeneration (PKAN) belongs to a group of hereditary neurodegenerative disorders known as neuroacanthocytosis (NA). This genetically heterogeneous group of diseases is characterized by degeneration of neurons in the basal ganglia and by the presence of deformed red blood cells with thorny protrusions, acanthocytes, in the circulation.

**Objective:**

The goal of our study is to elucidate the molecular mechanisms underlying this aberrant red cell morphology and the corresponding functional consequences. This could shed light on the etiology of the neurodegeneration.

**Methods:**

We performed a qualitative and semi-quantitative morphological, immunofluorescent, biochemical and functional analysis of the red cells of several patients with PKAN and, for the first time, of the red cells of their family members.

**Results:**

We show that the blood of patients with PKAN contains not only variable numbers of acanthocytes, but also a wide range of other misshapen red cells. Immunofluorescent and immunoblot analyses suggest an altered membrane organization, rather than quantitative changes in protein expression. Strikingly, these changes are not limited to the red blood cells of PKAN patients, but are also present in the red cells of heterozygous carriers without neurological problems. Furthermore, changes are not only present in acanthocytes, but also in other red cells, including discocytes. The patients’ cells, however, are more fragile, as observed in a spleen-mimicking device.

**Conclusion:**

These morphological, molecular and functional characteristics of red cells in patients with PKAN and their family members offer new tools for diagnosis and present a window into the pathophysiology of neuroacanthocytosis.

## Introduction

Panthothenate kinase-associated neurodegeneration (PKAN) belongs to the family of hereditary neurodegenerative disorders known as neuroacanthocytosis (NA), which includes chorea-acanthocytosis (ChAc), McLeod syndrome (MLS) and Huntington’s disease-like 2 (HDL2) [1]. NA is a genetically heterogeneous group of diseases, characterized by neurodegeneration, affecting mainly the basal ganglia and leading to progressive movement disorders, with cognitive and psychiatric features [1,2]. PKAN has been recently included in NA as a recessive NBIA disorder (neurodegeneration with brain iron accumulation), displaying clinical manifestations similar to those of NA, but characterized by the accumulation of iron in the basal ganglia [3].

One of the biological hallmarks of NA is the presence of acanthocytes, red blood cells (RBCs) with thorny protrusions, in the blood [1,2]. The association of acanthocytosis and neurodegeneration of the basal ganglia suggests a common pathogenic pathway, which can easily be explored by studying NA red cells. Changes in the structure and function of band 3, a key integral protein of the red cell membrane, as well as abnormalities in a band 3-regulating signaling network occupy a central position in our knowledge on the RBCs from NA patients [4,5]. However, the relation between the disease-causing mutations, the abnormal red cell shape, the structural changes and their effects on the function of the acanthocytic red cells remains mainly unknown.

In comparison with ChAc and MLS, only limited data are available on the characteristics of the RBCs of PKAN patients [3]. Only recently, a reduced response of acanthocytic PKAN erythrocytes to drug-induced endovesiculation has been described to suggest a perturbation of red cell membrane function in patients with PKAN [6].

Here, we describe for the first time a qualitative and semi‐quantitative morphological, structural and functional analysis of the RBCs from clinically diagnosed PKAN patients and their relatives.

Our results show the presence of morphological, structural and functional changes in RBCs not only of patients, but also in the RBCs of some of their relatives.

## Materials and Methods

### Design of the study and ethical considerations

**Fig 1:**
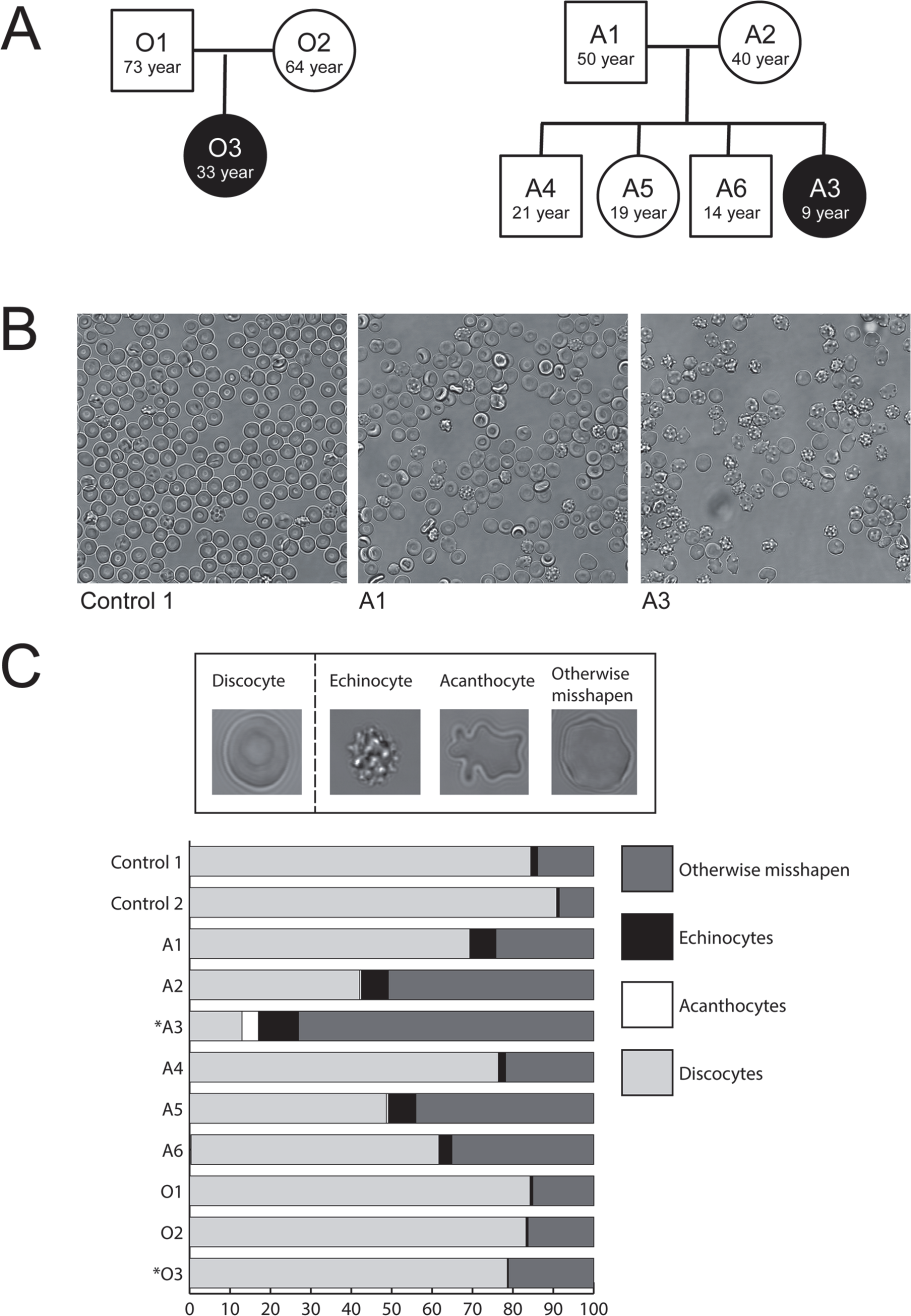
Pedigree and RBC morphology of the subjects in this study. (A) Patients who are clinically diagnosed with PKAN are indicated in black, the healthy relatives are indicated in white. (B) Representative pictures of blood films used for classification of cell shape. (C) Cells are classified in discocyte, echinocyte, acanthocyte or otherwise misshapen. The graph depicts the percentages of different cell morphologies in PKAN patients (marked with an asterisk) and their unaffected family members, compared to healthy donors (control 1+2) (see also S1 Fig for other PKAN families).

Blood was donated by healthy volunteers, patients and their relatives after written informed consent, using venipuncture and EDTA as anticoagulant. Control donors and patients had the same ethnic background. Patients were clinically diagnosed with PKAN [3], which could be confirmed by mutational analysis for some patients (S1 Fig). Family trees are shown in Fig 1A (family A and O) and S1 Fig for other PKAN patients and their family members (family C, N, UL, U), for which a complete set of analyses could not be obtained.

RBCs were isolated from 5–10 ml blood and separated from platelets and white blood cells using Ficoll (GE Healthcare, Waukesha, WI, USA) density centrifugation. The study was performed following the guidelines of the local medical ethical committees and in accordance with the declaration of Helsinki. As a part of the EMINA project, funded by E-Rare (40-41905-98-9005), this study was approved by the ethics committee of the Ludwig-Maximillius-Universität, Münich.

### Cell counts and classification

Cells were resuspended in Ringer solution (125 mM NaCl, 5 mM KCl, 1 mM MgSO_4_, 2.5 mM CaCl_2_, 5 mM glucose, 32 mM HEPES, pH7.4), poured into Lab-Tek chambered cover glass (Thermo Fisher Scientific, Rochester NY, USA), and random field pictures were taken for each sample. Counting and classification were performed on at least 400 cells by two independent operators, using the classifiers discocyte, echinocyte, acanthocyte and otherwise misshapen cell (Fig 1B). All microscopy was performed using a TCS SP5 confocal laser scanning microscope (Leica Microsystems, Mannheim, Germany) equipped with an HCX Plan-Apochromat 63X NA 1.2 water immersion lens.

### Plasma microparticle (MP) analysis

MP purification from plasma and analysis was performed as previously described [7]. MPs were stained with monoclonal anti-Glycophorin A-PE (clone KC16, Beckman Coulter, Fullerton, CA, USA) at a 1:100 dilution to specifically identify RBC-derived MPs.

### MP formation in vitro

RBCs were suspended in Ringer to a hematocrit of 4% and incubated for 4 or 24 hours at 37°C in a humidified incubator. MPs were isolated from the supernatant by centrifugation at 21,000xg for 30 minutes [8]. Counting and staining of MPs was performed as described previously [7], with staining for Band 3, using the K2N6B antiserum [8] at 1:500 dilution.

### Immunofluorescence

RBCs were fixed and stained as described previously [9]. For blocking purposes, 1% BSA was used. Primary antibodies and dilutions were mouse anti-stomatin (GARP-50) at a 1:100 dilution, mouse anti-band 3 N-terminal domain (B3-136, Sigma-Aldrich, St. Louis MO, USA) at a 1:100 dilution and mouse anti-β-spectrin (Acris, Herford, Germany) at a 1:50 dilution. The secondary antibody was goat anti-mouse Alexa 488 (Invitrogen, Carlsbad CA, USA) at a 1:1000 dilution. As internal controls, RBCs of one healthy control donor were stained with a rabbit antibody against the membrane domain of band 3 (IVF12, dilution 1:100 [10]) and these RBCs were mixed 1:1 with all patient, relative and all other healthy control samples. RBCs were imaged by confocal microscopy as described above. Image analysis was carried out using ImageJ version 1.45J. Cells were categorized by shape and total cell-associated fluorescence was calculated by adding the signals from all the planes composing the Z-stacks. From these data the mean fluorescence intensity (MFI) was measured per cell. In order to adjust for variance between different slides, the fluorescence of each sample was related to the same internal control.

### RBC membrane preparation and immunoblot analysis

RBC membranes were obtained by lysing one volume of packed RBCs in 10 volumes of ice-cold lysis buffer (5 mM Na_2_HPO_4_, pH 8.0 with protease inhibitors (cocktail tablet; Roche, Basel, Switzerland), 3 mM benzamidine and 1 mM Na_3_VO_4_) for 10 min on ice. The membrane fraction was washed repeatedly by centrifugation at 21,000xg for 10 min to remove free hemoglobin and solubilized 1:1 with Laemmli buffer (Bio-Rad Laboratories, Hercules CA, USA) supplemented with 100 mM β-mercaptoethanol for SDS-PAGE. SDS-PAGE was carried out according to Laemmli [11] on 10% polyacrylamide gels. Proteins were transferred to PVDF membranes using the iBlot system (Invitrogen, Carlsbad CA, USA). These membranes were blocked with Odyssey Blocking Buffer (LI-COR, Lincoln NE, USA) and probed with 1:2000 mouse anti-band 3 N-terminal domain (BIII-136, Sigma-Aldrich, St. Louis MO, USA), 1:5000 mouse anti-β-spectrin (Acris, Herford, Germany), or 1:10000 anti-β-actin (Sigma-Aldrich). As secondary antibodies 1:10000 goat anti-rabbit IgG-Alexa Fluor 680 (Invitrogen, Carlsbad CA, USA), and/or goat anti-mouse IgG-IRDye 800 (LI-COR, Lincoln NE, USA) were used. The blots were scanned with the Odyssey Infrared Imaging System (LI-COR, Lincoln NE, USA) and the images were analyzed using the Odyssey Software version 2.1.

### Microfluidics

RBC deformation within the microcapillaries was simulated with a microfluidic device with narrow, 7 μm width, channels as described previously [12]. Flow of cells was observed through a UPLFLN 100X, NA 1.3 oil immersion objective (Olympus, The Netherlands) using an optical microscope (IX71, Olympus) equipped with a high-speed CMOS camera (Phantom high speed camera, Vision Research, UK). In each run, a sequence of approximately 20,000 images was recorded.

### Spleen-mimicking device

RBC deformability was further assessed using a bead-sorting device that mimics the mechanical deformation that RBCs experience in the spleen, as described previously [13]. RBCs from the donor of interest were labeled with CFSE diacetate [14]. A 2% hematocrit suspension in Ringer with 1% BSA (6 ml), consisting of 5% labeled patient RBCs and 95% unlabeled RBCs from a healthy control donor, was passed through the bead-sorting device at a flow rate of 60 ml/h. Flow cytometry analysis of the upstream fraction, the retained fraction, and the downstream fraction was performed to determine the ratio of labeled versus unlabeled RBCs in each separate sample.

### Percoll separation

RBC fractionation according to cell density was performed using a discontinuous Percoll gradient consisting of six layers ranging from 40% Percoll to 80% Percoll, as described previously [15]. The RBCs were combined into four fractions: Fraction 1, 61% Percoll + 64.5% Percoll; Fraction 2, 67.5% Percoll; Fraction 3, 71% Percoll; and Fraction 4, 80% Percoll.

### HbA1c and HbF measurements

Red cell lysates were analyzed on an HA 8160 hemoglobin analyzer (Menarini Diagnostics, Italy) to determine the HbA1c and HbF concentrations.

## Results

### Abnormally shaped red cells are present in the blood of PKAN patients and their relatives

In order to acquire a better understanding of the morphological and functional characteristics of PKAN red blood cells, we performed a detailed morphological analysis of red cells from patients with PKAN as well as from their family members without neurological symptoms.

This analysis showed that the presence of acanthocytes was always accompanied by that of echinocytes and other misshapen cells (Fig 1B; see also S1 Fig). Although not all PKAN patients had acanthocytes, most patient’s blood samples contained a high percentage of morphologically abnormal RBCs. Furthermore, the blood of some relatives without neurological symptoms contained abnormally shaped red cells (e.g. family A in Fig 1B; see also family UL in S1 Fig).

**Fig 2:**
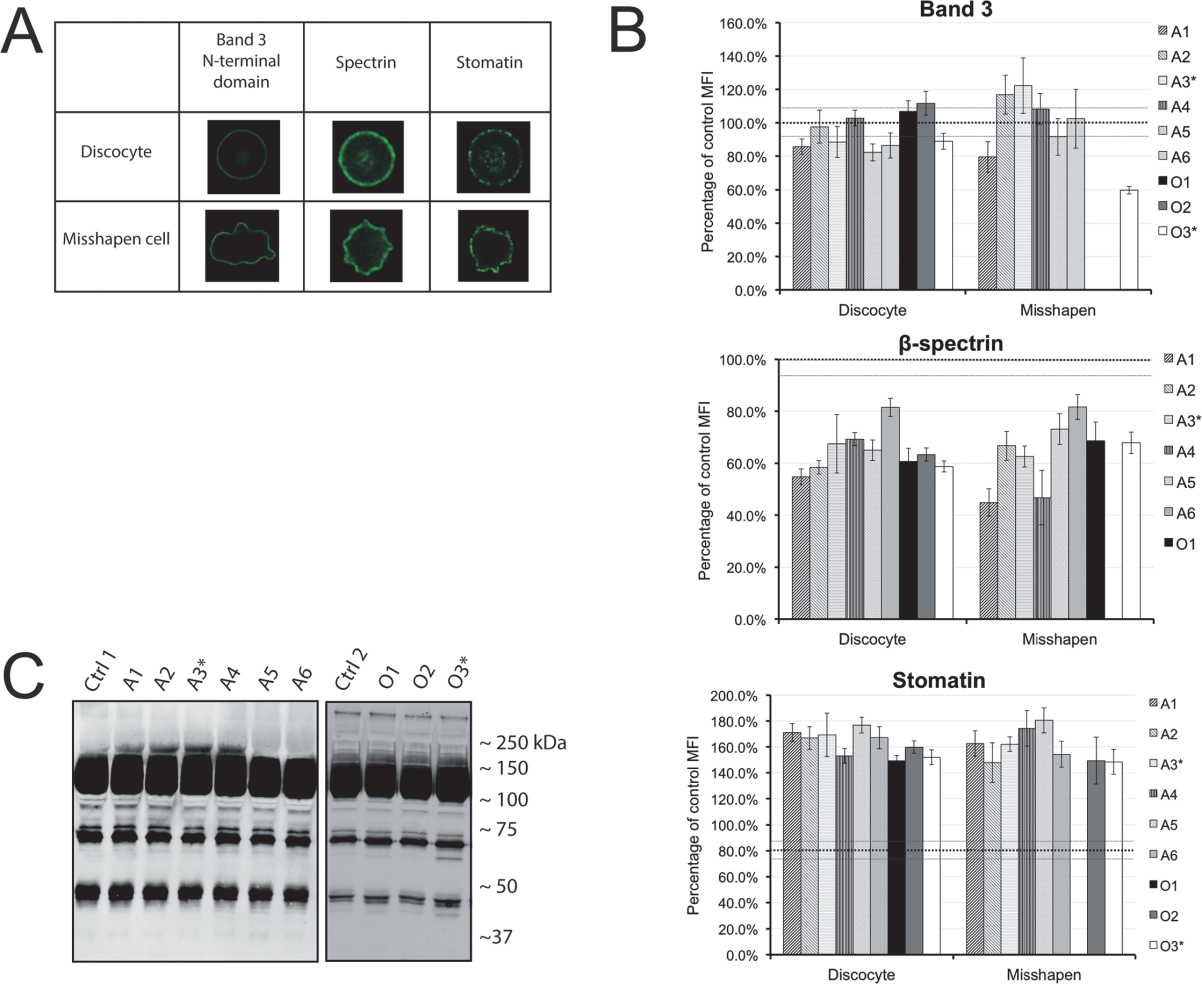
Quantitative and qualitative analysis of the main components of the membrane-cytoskeleton complex. (A) Confocal microscopy was used to categorize the red blood cells by their cell shape in discocyte versus misshapen cells. Immunofluorescence staining of band 3, spectrin and stomatin showed that the distribution of these proteins is similar in healthy looking cells and misshapen cells. (B) Quantitative image analysis of at least 25 cells was used to determine the mean fluorescent values of antibodies against band 3, β -spectrin and stomatin in relation to the control samples (dotted black line at 100%). Error bars represent the standard error. The grey dotted line depicts the standard error of the controls. (C) Band 3 in membrane fractions. Western blot staining with an antibody against the N-terminal domain of Band 3 (see also S2 Fig). Aberrant degradation patterns are seen in the 40 and 70 kDa area.

In order to obtain more insight into the molecular cause(s) underlying these aberrant morphologies, we evaluated the distribution and organization of the integral membrane protein band 3, the cytoskeleton protein spectrin, and the raft-associated stomatin with confocal microscopy. The total fluorescence intensity of the various antibody stainings was measured per cell and the cells were categorized by shape (Fig 2A). A quantitative analysis (see Materials and Methods) showed no differences in the amounts of band 3 in the RBCs of the controls, patients or relatives. However, the spectrin signal was decreased, whereas the stomatin signal was increased in the RBCs of PKAN patients and their relatives (Fig 2B). These changes were present in all red cells, independent of their shape. Immunoblots showed aberrant degradation of band 3 in some patients and their relatives (Fig 2C family O; S2 Fig family N, C), but normal stomatin and spectrin staining patterns (S3 Fig). Taken together, these data suggest perturbation in membrane protein organization, rather than protein abundance, in the RBCs of PKAN patients.

### Increased numbers of RBC-derived microparticles are present in PKAN patients with acanthocytes

**Fig 3:**
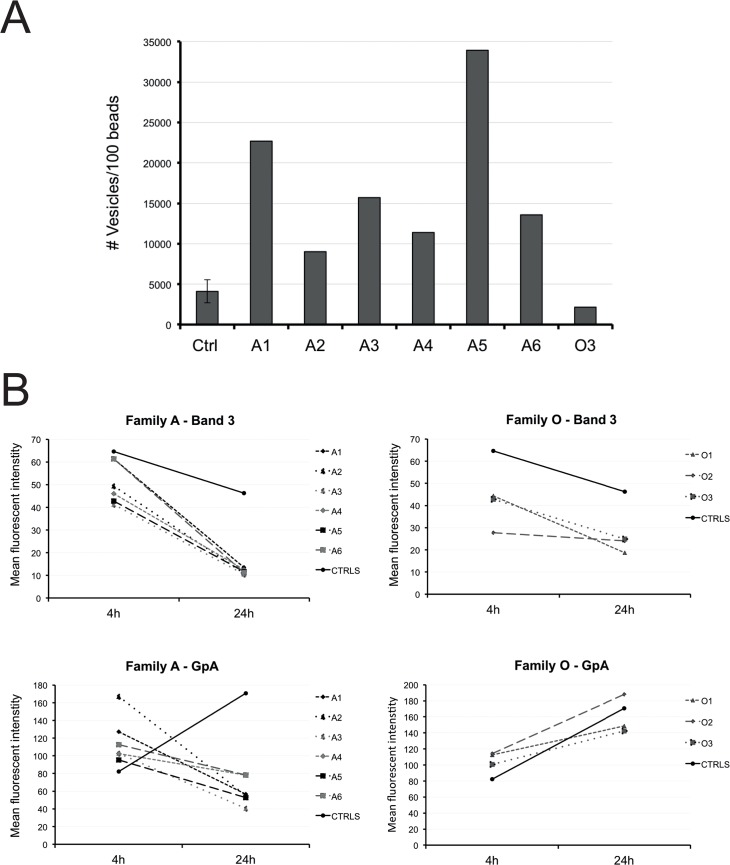
Microparticle formation by PKAN RBCs as characterized by flow cytometry. (A) The amount of RBC MPs in plasma. (B) Membrane composition of in vitro generated MPs. Band 3 and glycophorin A (GpA) was measured on MPs collected after 4 and 24h incubation of the RBCs in Ringer solution at 37 °C.

Since altered membrane organization has been described to favor MP formation during physiological aging and in various hereditary red cell disorders [16], we evaluated the amount of RBC-derived MPs in the blood of PKAN patients. The numbers of these MPs in the plasma were increased in all members of family A, but not in family O (Fig 3A). Analysis of the composition of MPs produced in vitro showed that the concentration of band 3 decreased for all MPs with time. Interestingly, the concentration of glycophorin A-positive vesicles increased for controls and O family with time, but decreased for family A (Fig 3B), suggesting an association between the number of MPs that are generated *in vitro* and their composition (Fig 2A).

### PKAN red cells are less resistant to deformation in a spleen-mimicking device

**Fig 4:**
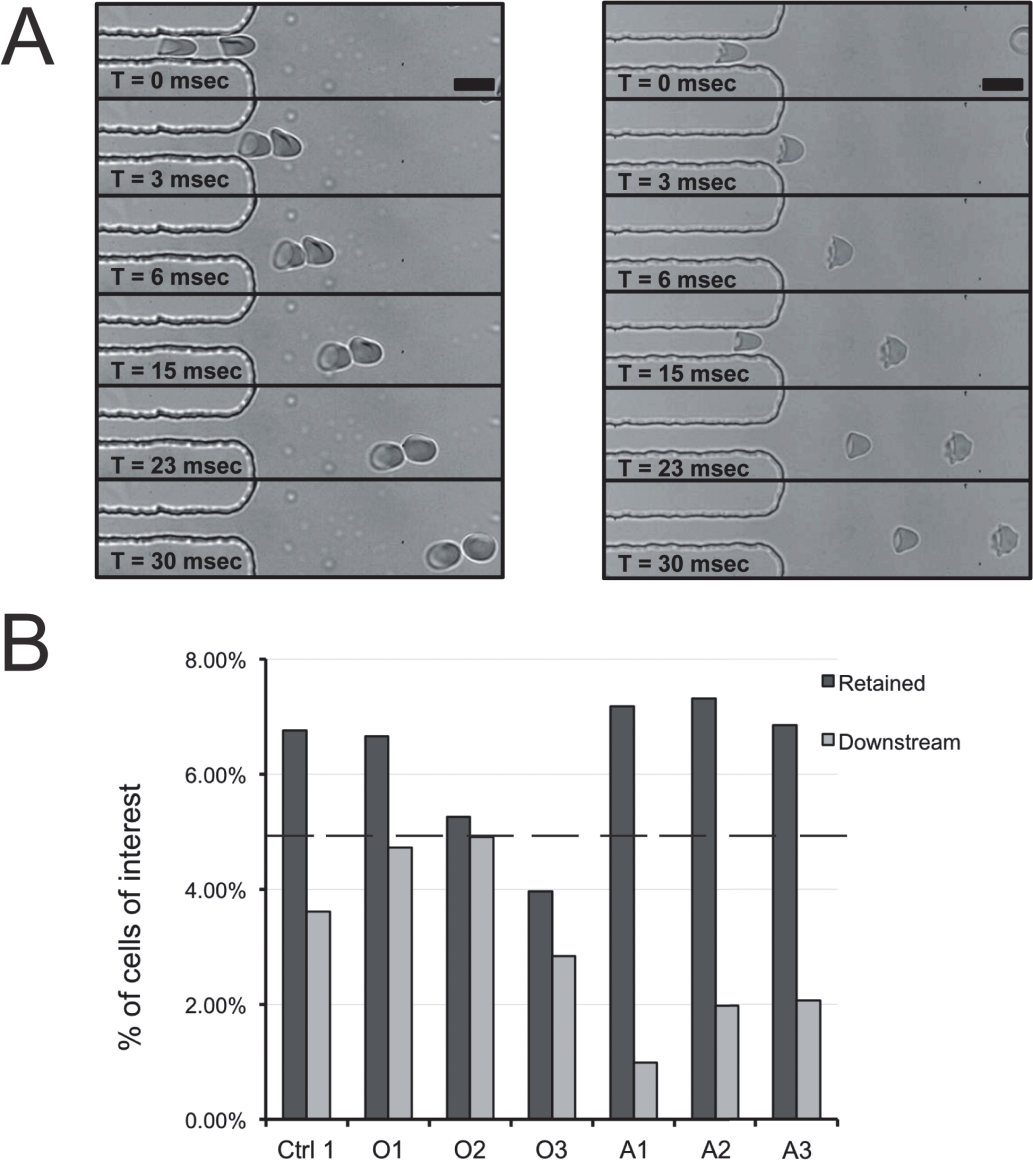
Deformability and fragility studies of RBCs. (A) RBC deformability and relaxation capacity was studied with a microfluidic device [12]. Scale bar 10 μm. (B) Deformability and fragility were also measured with a spleen-mimicking device [13]. The cells of interest were stained with CFSE, mixed with RBCs of a control donor, and passed through a bed of metal beads [13,14]. Retention was measured by the number of cells that did not pass through the bead layer. The percentages of CFSE-positive cells in the retained and downstream sample were compared to that in the upstream sample (set at 5%, depicted as a dotted line in panel B).

One of the main functional features of RBCs is their capacity to deform in the microcirculation and in the narrow sinusoidal space of the spleen [13,17]. We used microfluidics technology to simulate the physiological conditions that RBCs encounter within the microcapillaries, and to analyze deformability at the single cell level [12]. We found no major changes in red cell PKAN deformability and relaxation compared to healthy red cells, notwithstanding their morphological abnormalities (Fig 4A). Within the narrow channels, acanthocytes were only discernible by minor irregularities, but upon exiting the channels, they immediately relaxed back into their typical shape with thorn-like protrusions (Fig 4A, see also S1 Video). However, the ability of PKAN RBCs to undergo a more extreme deformation stress, as analyzed with a spleen-mimicking device, was severely reduced compared to healthy controls (Fig 4B). RBCs from patient A3 and from his parents were much more retained than control red cells. The RBCs of patient O3 were also more fragile than healthy red cells (Fig 4B); analysis of the retained and downstream samples indicated that a considerable number of the patients' cells was lost, suggesting a high susceptibility to mechanical stress leading to cell lysis.

### Hemoglobin composition indicates altered RBC homeostasis in PKAN patients

**Fig 5:**
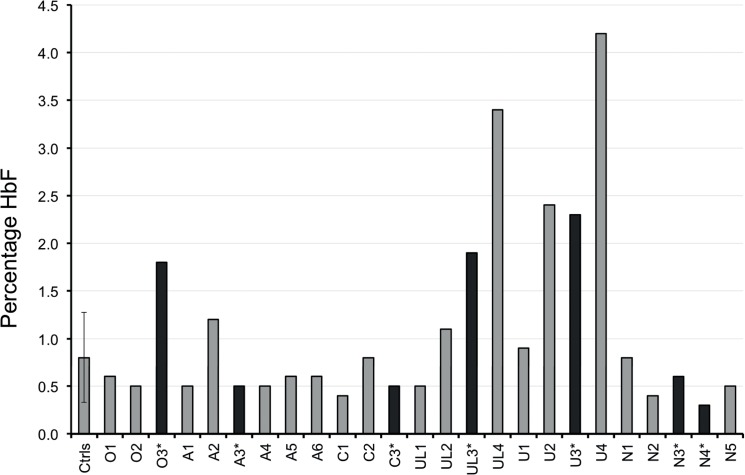
Measurements of fetal haemoglobin (HbF) in red cells of PKAN patients and their relatives. Black, patients; grey, controls (Ctrls) and family members. The control value is the mean of 6 control samples. The error bar depicts the standard deviation.

Since altered MP formation and increased retention in the spleen could affect RBC lifespan, we measured HbA1c in PKAN and healthy red cells as an indirect, independent parameter of red cell survival [18,19]. Routine hematological parameters, including reticulocyte counts and HbA1c values of PKAN patients and their relatives were within in the normal range. This provided no indications for a significant reduction in RBC lifespan. However, the RBCs of several PKAN patients and their family members contained increased HbF levels (Fig 5A). These findings may indicate alterations in erythropoiesis in patients with PKAN [20].

## Discussion

Here we report an extensive morphological and functional description of the red cells of patients with PKAN and their relatives, which presents a unique opportunity to investigate the pathophysiology of this rare neurodegenerative disorder.

Our data suggest that the acanthocytic shape is only one of the various possible abnormal morphologies of RBCs in patients with PKAN. The presence of increased levels of misshapen red blood cells in the blood of relatives without any neurological symptoms indicates that perturbation of membrane organization may not be limited to the homozygous state of the disease. Together with the immunofluorescence and immunoblot data, this suggests that the membrane organization of RBCs may be more sensitive to the presence of pathological NA proteins than that of neuronal cells. An extensive morphological analysis of the red cells of patients with other forms of NA and their relatives is required to test the general applicability of this conclusion. At present, we cannot exclude that the misshapen red cells represent a stage before the acanthocytes, and that the transition occurs as a consequence of aging and/or stress experienced during their life [4]. However, this theory is not supported by the HbA1c levels, which were not significantly different for acanthocyte-rich fractions. Instead, our finding that HbF is increased in RBCs from patients with NA may suggest that acanthocytes are already formed during erythropoiesis. Increased HbF levels have been associated with altered erythropoiesis [19,21]. Indeed, a mild hemolytic anemia is described in many NA patients [22], which could induce stress erythropoiesis resulting in increased HbF [23]. Thus, our data may imply that especially erythropoiesis is sensitive to pantothenate kinase-associated alterations in coenzyme A synthesis.

The decreased spectrin content in PKAN RBCs as detected by our immunofluorescence analysis may be due to a PKAN-related reduction in the spectrin content or to a reduced accessibility of epitopes for the anti-spectrin antibody. Discrepancies between immune assays and other semi-quantitative methods have been noted before in molecular studies on red blood cells and acanthocytosis [4,24]. The immunoblots showed no indications for degradation, but any breakdown products may have been degraded and removed from the cells [4,25]. An altered epitope accessibility may be related to changes in phosphorylation-sensitive protein-protein interactions underlying abnormal cell morphology [24,26]. The available data do not enable us to distinguish between an innate lower spectrin content in newly matured red blood cells and a loss of spectrin during their stay in the circulation. Since these alterations were not associated with the number of acanthocytes, it is likely that all red cells in PKAN patients present abnormal features, which result in abnormally shaped red cells and acanthocytes only in a fraction of the cells.

The changes in staining for the raft marker stomatin, possibly in close association with altered microparticle generation (Salzer et al., 2002), constitute other indications for a perturbation of the membrane organization in PKAN red blood cells. Since misshapen red cells are generally associated with decreased red cell deformability [27], we analyzed the deformation of PKAN red cells in a microfluidic device. The deformability of discocytes and misshapen cells from PKAN patients was similar to that of red cells from healthy controls. However, the decreased deformability and/or the increased fragility of PKAN red cells in the spleen-mimicking device suggest an anomaly in the intrinsic membrane deformability of PKAN red cells (Fig 4B) This fragility could explain the slight hemolytic anemia in NA patients [22].

In conclusion, we report for the first time an extensive characterization of red cells from PKAN patients and family members. We showed increased numbers of misshapen red cells together with acanthocytes in PKAN subjects, but also in some of their relatives without neurological symptoms. The morphological abnormalities were associated with indications for perturbation in cytoskeleton and lipid bilayer organization, possibly underlying altered microparticle formation and reduced deformability in the spleen.

## Supporting Information

S1 Fig Pedigrees and red cell morphology of other PKAN families. (A) Patients that are clinically diagnosed with PKAN are indicated in black, the healthy relatives are indicated in white. PKAN diagnosis could be confirmed by mutation analysis for patient N3 and N4. (B) The graph depicts the percentages of various RBC categories in PKAN patients (marked with an asterisk) and their family members, compared to healthy donors (control 1, 2 and 3)
(EPS)

S2 Fig Band 3 in membrane fractions of several PKAN patients. Western blot staining with antibody against N-terminal domain of Band 3, as described in the Material and Methods section. Aberrant degradation patterns were found between the 35 and the 70 kDa area.
(EPS)

S3 Fig Spectrin and stomatin in membrane fractions of PKAN patients and their family members. Western blot staining with antibody against beta-spectrin and stomatin, as described in the Material and Methods section.
(EPS)

S1 Video Deformation and relaxation of RBCs of a PKAN patient within microfluidic channels mimicking the microcirculation in vivo. (AVI)
